# Repurposing Oxfendazole for Onchocerciasis: Population Pharmacokinetics of a Tablet Formulation in Healthy African Adults

**DOI:** 10.1002/psp4.70189

**Published:** 2026-02-09

**Authors:** Frauke Assmus, Ayorinde Adehin, Richard M. Hoglund, Gloria Nyaulingo, Hussein Mbarak, Said Jongo, Eveline Ackermann, Elisabeth Reus, Jennifer Keiser, Fabiana Barreira Da Silva Rocha, Sabine Specht, Ivan Scandale, Joel Tarning

**Affiliations:** ^1^ Mahidol Oxford Tropical Medicine Research Unit, Faculty of Tropical Medicine Mahidol University Bangkok Thailand; ^2^ Centre for Tropical Medicine and Global Health, Nuffield Department of Medicine University of Oxford Oxford UK; ^3^ Ifakara Health Institute Bagamoyo Tanzania; ^4^ Swiss Tropical and Public Health Institute Allschwil Switzerland; ^5^ University of Basel Basel Switzerland; ^6^ Drugs for Neglected Diseases initiative Geneva Switzerland; ^7^ Infectious Diseases Data Observatory University of Oxford Oxford UK

**Keywords:** healthy African volunteers, nonlinear mixed effects modeling, onchocerciasis, oxfendazole, population pharmacokinetics, tablet

## Abstract

Global efforts to eliminate onchocerciasis are hampered by the lack of a macrofilaricidal drug capable of killing adult parasites. Oxfendazole, a veterinary anthelminthic, exhibits macrofilaricidal activity and holds promise to shorten treatment durations. Phase 1 studies in healthy Caucasian adults demonstrated favorable pharmacokinetics and safety using a veterinary oral liquid formulation. More recently, a Phase 1 bioavailability trial (NCT04920292) evaluated a field‐applicable tablet formulation in healthy African adults. This study presents a secondary analysis to (i) characterize the population pharmacokinetics of oxfendazole and its major metabolites in healthy African adults receiving the tablet formulation and (ii) propose a dosing regimen for Phase 2 evaluation in patients with onchocerciasis. Thirty healthy African adults were enrolled, and plasma concentration–time profiles of oxfendazole, fenbendazole, and oxfendazole sulfone were obtained from 24 participants who received oxfendazole (8 per dose group: 100 mg single dose, 400 mg single dose, 400 mg once daily for 5 days). All cohorts were pooled and analyzed using nonlinear mixed effects modeling. Oxfendazole absorption was best described by first‐order kinetics with first‐pass metabolism. Dose‐limited bioavailability was evident. Disposition was best described by one‐compartment models with linear elimination. Simulations suggested that 400 mg once daily (or 50 mg twice daily) for 5 days is required to achieve putative exposure targets (> 200 ng/mL for 5 days), with low risk of safety concerns. The population pharmacokinetic model adequately described oxfendazole pharmacokinetics in healthy African adults and supports dosing selection for future clinical trials.

**Trial Registration:** ClinicalTrials.gov Identifier: NCT04920292

## Study Highlights

- What is the current knowledge on the topic?
  ○ Oxfendazole is a veterinary anthelminthic with macrofilaricidal activity against a broad range of parasitic worms. Following a repurposing strategy, it is currently under clinical development for the treatment of human helminth infections, including onchocerciasis. Earlier Phase 1 studies in healthy Caucasian volunteers receiving a liquid formulation showed favorable pharmacokinetics and safety. To support broader use in endemic settings, a field‐adapted tablet formulation was developed.
- What question did this study address?
  ○ This study characterized the population pharmacokinetic properties of the oxfendazole tablet in healthy African adults and identified suitable dosing regimens for Phase 2 trials in onchocerciasis patients.
- What does this study add to our knowledge?
  ○ This is the first population pharmacokinetic analysis of oxfendazole in African adults using the tablet formulation. The model incorporated first‐pass metabolism and dose‐limited bioavailability and revealed substantial variability in drug exposure. Simulations suggested that a 400 mg once‐daily or 50 mg twice daily dose of oxfendazole over 5 days is likely to achieve proposed target exposures, with low risk of safety concerns.
- How might this change drug discovery, development, and/or therapeutics?
  ○ The developed model provides a quantitative basis to guide dose selection of oxfendazole in future clinical trials. Despite variability in exposure and the need for formulation optimization, the findings support continued development of oxfendazole as a macrofilaricidal drug for onchocerciasis.

## 1 Introduction

Onchocerciasis (“river blindness”) is a neglected tropical disease caused by the filarial worm *Onchocerca volvulus*. As of 2017, an estimated 20.9 million people worldwide were infected, and at least 220 million required preventive chemotherapy [1, 2]. The largest burden of disease is concentrated in 31 African countries, mainly in sub‐Saharan Africa, where onchocerciasis still poses an important public health challenge. Clinical manifestations include debilitating skin disease, visual impairment, and permanent blindness [2]. Onchocerciasis is the world's second leading infectious cause of blindness and is increasingly associated with high rates of epilepsy in regions with ongoing transmission [1, 3, 4].

Although onchocerciasis has been targeted for elimination as a public health problem by 2030 [5], the lack of a macrofilaricidal drug capable of killing adult *O. volvulus* worms remains a major obstacle [6, 7]. The cornerstone of current elimination efforts is mass drug administration (MDA) with ivermectin, which effectively clears microfilariae but fails to kill adult worms [8]. Consequently, repeated (bi)annual treatment over the 10–15 years life span of adult worms is required to interrupt transmission. Another limitation of ivermectin is the risk of severe adverse events in individuals with high *Loa loa* microfilarial loads [9, 10]. Areas co‐endemic with *Loa loa* are therefore often excluded from MDA programs, leaving many patients untreated. Transmission modeling suggests that ivermectin alone may be insufficient to achieve the 2030 elimination targets [11, 12], and reports of suboptimal response and emerging resistance are increasingly concerning [13]. A macrofilaricidal treatment is urgently needed.

Oxfendazole is a benzimidazole anthelminthic with broad‐spectrum activity against various intestinal and tissue dwelling helminths [14]. It has a longstanding history of veterinary use for the control of lung, stomach, and intestinal worms in livestock [15, 16]. Oxfendazole acts by inhibiting microtubule polymerization in helminths, leading to the disintegration of crucial cellular structures [17, 18].

Its efficacy against both larval and adult stages of parasitic worms has been demonstrated across multiple animal species [14, 19, 20]. Notably, oxfendazole has shown macrofilaricidal activity in preclinical models for human filariasis, including the *Onchocerca gutturosa* adult worm and the *Litomosoides sigmodontis* mouse model [21]. Its potency was recently confirmed in a novel *O. ochengi* gerbil model—closely related to *O. volvulus*—where it effectively inhibited adult worm motility [22]. Importantly, no direct microfilaricidal effect has been observed in vivo [21], potentially reducing the risk of severe adverse events in individuals co‐infected with *Loa loa*. The metabolites, fenbendazole and oxfendazole sulfone, have also shown activity against adult *O. gutturosa*, although with lower potency than the parent compound [21]. Fenbendazole is itself approved as a broad‐spectrum veterinary drug for treating gastrointestinal parasites in both large and small animals.

Apart from its promising efficacy [14], a comprehensive nonclinical safety review provided further support to repurpose oxfendazole for human use [23]. The Oxfendazole Development Group (ODG) [24] conducted first‐in‐human (FIH) single and multiple ascending dose studies (SAD, MAD) in healthy, primarily Caucasian volunteers using a veterinary oral liquid formulation [25, 26]. Oxfendazole was well tolerated in these studies and exhibited nonlinear pharmacokinetics (PK), likely due to solubility‐limited absorption.

To advance clinical development, a field‐applicable, immediate‐release tablet formulation was developed and evaluated in a Phase 1 study in healthy African adults residing in Tanzania, a filariasis‐endemic country. Safety, tolerability, and PK based on noncompartmental analysis (NCA) from this study are reported separately [27].

In the present study, we used data from this Phase 1 trial to characterize the population PK properties of oxfendazole and its major metabolites in healthy African adults. The results of this analysis, along with simulations of different dosing scenarios, are presented to inform dose selection for future clinical trials.

## 2 Methods

### 2.1 Clinical Study Design

This population PK analysis is based on data from the HELP‐OFZ study, a Phase 1 study conducted in 2022 at the Bagamoyo Clinical Trial Facility in Tanzania. The study was sponsored and led by the Swiss Tropical and Public Health Institute (Swiss TPH) and conducted in partnership with the Ifakara Health Institute (IHI) and the Drugs for Neglected Diseases *initiative*. Details on the study design, inclusion criteria, and drug formulation are reported elsewhere [27]; a brief summary is provided below.

HELP‐OFZ was a randomized, placebo‐controlled, double‐blind, single center Phase 1 study in 30 healthy African volunteers. Participants were eligible if they were healthy adults (aged 18–45 years), male or female (nonpregnant, nonbreastfeeding), residing in Tanzania, with a body mass index between 18.0 and 29.9 kg/m^2^, and no evidence of parasitic or chronic infections.

Oxfendazole was administered orally in the fasted state as an immediate‐release tablet formulation. Participants were assigned to one of three cohorts: 100 mg single dose, 400 mg single dose, or 400 mg once daily for 5 consecutive days. In each cohort, 10 participants were randomized to receive either oxfendazole (8 per cohort) or matching placebo (2 per cohort).

### 2.2 Ethics Statement

The study was conducted in compliance with the Declaration of Helsinki, ICH‐GCP guidelines, and national regulatory requirements. Ethical approval was obtained from the IHI Institutional Review Board (IHI/IRB/No: 48‐2021) and the National Health Research Ethics Committee in Tanzania (IMR/HQ/R.8a/Vol. IX/3894). Additionally, the Independent Ethics Committee (Ethikkommission Nordwest‐ und Zentralschweiz [EKNZ]) in Switzerland issued a positive statement (AO_2021_00043). All participants provided written informed consent prior to enrollment.

### 2.3 Blood Collection and Quantification

Frequent blood samples (3 mL) were collected in all volunteers. In the single‐dose cohorts (cohorts 1 and 2), samples were collected at 0 (predose), 1, 2, 3, 4, 6, 8, 10, 12, 24, and 48 h after drug administration. In the multiple dose cohort (cohort 3), samples were collected at 0 (predose), 1, 2, 3, 4, 8, and 12 h after administration of the first dose (day 1). On days 2 and 3, blood samples were collected at predose and 2 h after administration of each dose, while only predose blood samples were taken on day 4. From day 5 onwards, blood samples were collected at predose and 1, 2, 3, 4, 8, 12, 24, 48, and 72 h after the last dose. The exact sampling times were recorded and used for population PK modeling.

Plasma concentrations of oxfendazole and its metabolites, fenbendazole and oxfendazole sulfone, were quantified using a validated high‐performance liquid chromatography tandem mass spectrometry (HPLC‐MS/MS) method [27]. The lower limits of quantification (LLOQ) were 2 ng/mL for oxfendazole, and 1 ng/mL for both metabolites. Further details are provided in Table S1.

### 2.4 Population Pharmacokinetic Analysis

Plasma concentrations of oxfendazole and its metabolites from the 24 participants on active treatment were pooled across all cohorts and analyzed simultaneously using nonlinear mixed effects modeling in NONMEM (v7.4; Icon Development Solution, Ellicott City, MD, USA). Prior to analysis, plasma concentrations were converted to molar units and transformed into their natural logarithm. Concentrations below the LLOQ were omitted from analysis (overall, 8.4% were below the quantification limit, BQL); this approach was considered adequate, as no model misspecification related to the fraction of censored observations was observed.

Throughout the model development process, the first‐order conditional estimation method with interactions (FOCE—I) was used. Automation and diagnostics were facilitated by the use of Pirana (v2.9.9), Pearl‐speaks‐NONMEM (PsN v5.2), R (v4.2.2) and TIBCO Spotfire (v 11.3.0). Discrimination between two competing, nested models was based on objective function values (OFV), with the difference in OFV being equivalent to a log‐likelihood ratio test. A significant improvement between two hierarchical models was indicated by a decrease in OFV by at least 3.84 (*p* < 0.05, one degree of freedom difference).

A drug‐metabolite model was developed under the assumption that oxfendazole clearance was split equally between conversion to fenbendazole and oxfendazole sulfone (50% each), in order to maintain structural identifiability. One‐ and two‐compartment models were evaluated to describe the structural disposition of each drug. Different absorption models were explored, including first‐order absorption models with and without lag time, as well as transit‐compartment absorption models and first‐pass metabolism. Relative oral bioavailability (*F*) was fixed to unity for the population, with an estimated inter‐individual variability (IIV) incorporated into the base model.

The best performing structural model was carried forward to subsequent covariate model building. Body weight (standardized to a body weight of 70 kg) was implemented a priori as an allometric function on all clearance (exponent 0.75) and volume (exponent 1) parameters. Dose (in mg, categorical), sex (categorical) and age (continuous) were investigated as covariates on all PK parameters by a stepwise covariate modeling approach. In the forward step, covariates were included at a statistical significance level of *p* = 0.05 (ΔOFV = −3.84), followed by a more stringent backward elimination step (*p* = 0.001, ΔOFV = −10.83).

In the final model, dose‐limited bioavailability was implemented as a continuous function, according to [28], Equation (1):

(1a) $$\mathrm{Cov}={\left(\text{Dose}/34.7\mathrm{mg}\right)}^{-0.541}$$

(1b) $$F_{i}=F\times \mathrm{Cov}\times e^{n_{i,ϴ}},$$

where Dose is the administered dose (in mg). The coefficients −0.541 and 34.7 mg (reference dose) have been reported previously in a SAD study, where oxfendazole was administered as an oral suspension at doses ranging from 0.5 to 60 mg/kg [28]. *F*_*i*_ is the individually estimated fraction of dose that was absorbed (for the *i*th subject), and *F* is the population mean estimate thereof (fixed to 1), with *η*_*i*,ϴ_ denoting the IIV for *F*.

IIV and interoccasion variability (IOV) were implemented using an exponential error model. IOV was investigated on absorption parameters, treating each drug administration as a separate occasion [29]. Residual unexplained variability was implemented as an additive error on the log‐transformed observed concentrations (equivalent to an exponential residual error on the original scale), separately estimated for each compound.

Model performance was evaluated through goodness‐of‐fit (GOF) plots and prediction‐corrected visual predictive checks (VPCs). Bootstrapping (*n* = 1000 resampled bootstrap datasets) was performed to evaluate model robustness and to obtain parameter precision estimates, including relative standard errors (RSE) and 95% confidence intervals (CIs) for the final model.

The following secondary PK parameter estimates were derived from the observed plasma concentration—time profiles: cumulative area under the concentration‐time curves from time zero to infinity (AUC_∞_), peak plasma concentration (*C*_MAX_), total time above a putative target concentration of 200 ng/mL oxfendazole (T > C_TARGET_), time to reach *C*_MAX_ (*T*_MAX_) and terminal elimination half‐life (*t*_1/2_).

### 2.5 Simulations of Dosing Scenarios

Stochastic simulations, based on the final population PK model, were performed in NONMEM to evaluate various oxfendazole dosing regimens, administered as an immediate‐release tablet formulation. Dosing regimens included once‐daily doses of 12.5, 25, 50, 100, 200, 400, and 800 mg oxfendazole for 5 consecutive days, and twice‐daily doses of 6.25, 12.5, 25, 50, 100, 200, and 400 mg oxfendazole (every 12 h) for the same 5‐day period.

Simulations were performed for 1000 adults per dosing regimen with a body weight of 60 kg, taking dose‐limited bioavailability and estimated variability into account. In addition, target attainment analyses were performed across a range of adult body weights (42–75 kg), spanning the minimum and maximum body weights of the study population. From the simulated plasma concentration–time profiles, *C*_MAX_, AUC_∞_, and T>C_TARGET_ were derived for oxfendazole.

Target attainment analysis focused on oxfendazole only, as no defined target concentrations are available for its metabolites. Although the metabolites are pharmacologically active, their potency is lower [21], and their plasma exposures were approximately 8 to 9‐fold (oxfendazole sulfone) and over 40‐fold (fenbendazole) lower than that of oxfendazole.

Exposure targets for oxfendazole were defined as ≥ 5 days total time above 200 ng/mL in plasma, for at least 90% of the population (rationale provided in the Discussion). A comprehensive target attainment analysis was performed for a range of C_TARGET_ values (100–2000 ng/mL). Probability of target attainment (PTA) was calculated as the proportion of simulated individuals reaching the defined exposure targets. Target attainment analysis and graphical representations were done in R (v4.2.2).

## 3 Results

### 3.1 Study Population

A total of 30 healthy adults were enrolled in the HELP‐OFZ study. Of these, 24 participants received oxfendazole; eight in each of the three treatment arms: 100 mg single dose, 400 mg single dose, and 400 mg once daily for 5 days. The remaining six participants received placebo, with two assigned to each treatment arm.

Demographic and baseline laboratory characteristics of all 30 study participants are reported in the accompanying manuscript [27]. Key baseline demographic characteristics for the 24 subjects included in the population PK analysis are summarized in Table S2. All were healthy adults (age range: 19–41 years) of Black African ethnicity residing in Tanzania. The PK analysis population included 14 males (58.3%) and 10 females (41.7%), with a median body weight of 58 kg (min to max range: 42–75 kg). Demographic parameters appeared similar across cohorts.

### 3.2 Pharmacokinetic Data

The 24 participants included in the population PK analysis contributed a total of 984 post‐dose plasma concentration measurements—328 each for oxfendazole, fenbendazole, and oxfendazole sulfone. Of these, 901 PK samples were above the LLOQ (8.4% were BQL). All samples of oxfendazole and oxfendazole sulfone were quantifiable. For fenbendazole, 83 plasma concentrations (25.3%) were BQL, primarily during the formation phase; only 2 of 328 samples (0.6%) were BQL during the elimination phase. Notably, in one subject in the 400 mg/single dose cohort, all fenbendazole plasma levels were below the LLOQ.

**FIGURE 1:**
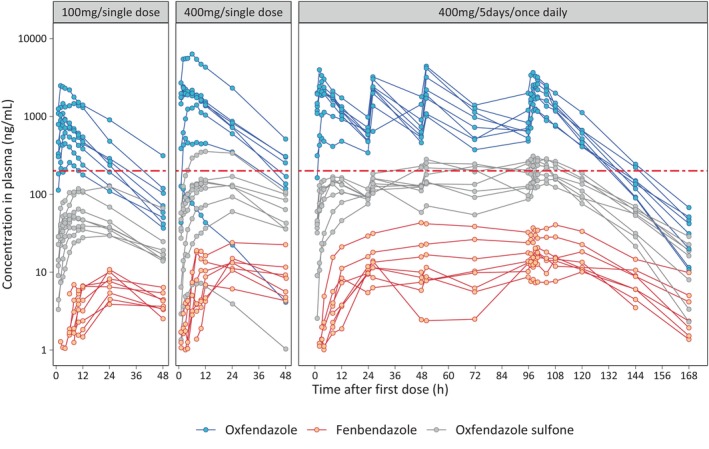
Plasma concentration–time profiles of oxfendazole and metabolites across cohorts. Individual plasma concentration–time profiles of oxfendazole, fenbendazole, and oxfendazole sulfone in healthy African adults (*n* = 8 per cohort) following administration of oxfendazole as a single 100 mg dose (cohort 1), single 400 mg dose (cohort 2), or 400 mg once daily for 5 days (cohort 3). For fenbendazole, all PK samples from one subject in cohort 2 were below the LLOQ and are not shown (*n* = 7). The horizontal red line represents the putative oxfendazole target concentration (C_TARGET_ = 200 ng/mL).

Individual plasma concentration–time profiles for oxfendazole, fenbendazole, and oxfendazole sulfone across the different dosing cohorts are shown in Figure 1. For two individuals, an inconsistency in concentration measurements at the 6‐h time point suggested a potential sample mix‐up. To address this, concentration values at that time point were swapped between the two individuals. Figure 1 reflects the corrected data; both the original and corrected profiles are shown in Figure S1.

### 3.3 Population Pharmacokinetic Model

**FIGURE 2:**
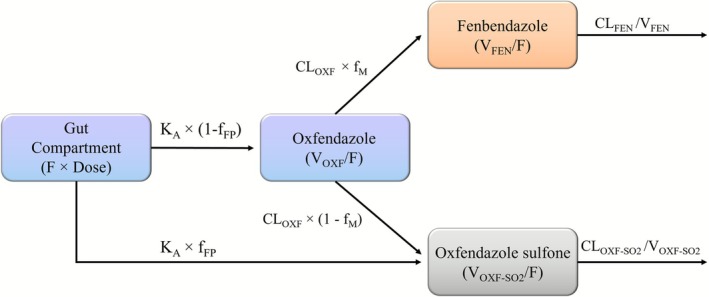
Graphical representation of the final model describing the pharmacokinetics of oxfendazole and its metabolites. The model incorporates a first‐pass effect, with an estimated fraction of the absorbed oral oxfendazole (f_FP_) converted into oxfendazole sulfone during first‐pass hepatic metabolism, while the remaining fraction (1 ‐ f_FP_) is absorbed unchanged. F denotes relative oral bioavailability of oxfendazole, K_A_ is the first‐order absorption rate constant, V/F is the apparent central volume of distribution, and CL/F is the apparent elimination clearance. Fractions of oxfendazole clearance attributed to metabolism to fenbendazole (FEN) and oxfendazole sulfone (OXF‐SO2) are denoted as f_M_ (set to 0.5) and (1 ‐ f_M_), respectively.

A graphical representation of the final structural model and its parameterization, describing the plasma concentration data for oxfendazole and its metabolites, is shown in Figure 2.

Oxfendazole plasma concentration–time profiles were best described by a first‐order absorption model with first‐pass metabolism to oxfendazole sulfone, followed by a one‐compartment disposition model. Elimination from the central compartment was described by a first‐order process, assuming that 50% of oxfendazole clearance each was attributed to the formation of fenbendazole and oxfendazole sulfone. Adding the first‐pass effect significantly improved the model fit compared to an absorption model without presystemic metabolism (∆OFV = −155.9). Without a first‐pass effect, the model yielded implausibly high absorption rate constants (K_A_ = 71.9 h^−1^). Incorporating first‐pass metabolism produced more realistic K_A_ estimates and was integrated in the base model. A flexible transit‐compartment absorption model did not further improve the fit and was discarded.

One‐compartment disposition models were implemented for oxfendazole and both metabolites. Two‐compartment disposition models were also tested but no significant improvement was observed for fenbendazole. Although two‐compartment disposition models for oxfendazole and oxfendazole sulfone improved the model fit (∆OFV = −13.9 and −21.3), they produced high variability (> 85%–798% IIV), poor precision (> 400% RSE), and unreasonably long elimination half‐lives (> 32 days). Therefore, one‐compartment disposition models were retained and carried forward.

Body weight was implemented a priori as an allometric function on all volume and clearance parameters with minimal impact (∆OFV = 0.7). A trend toward lower relative bioavailability at higher doses was observed (∆OFV = −4.3), indicating a 52% reduction in *F* when increasing the dose from 100 to 400 mg. This trend was significant in forward inclusion, but not backward elimination. None of the other covariates were retained in the final model after backward elimination.

To allow for simulations beyond the studied doses and to enhance model applicability, the impact of dose on the extent of absorption was incorporated using a previously published function, despite the lack of significance in the backward elimination step. A prior SAD study evaluated oxfendazole as an oral suspension over a wide dose range (0.5–60 mg/kg) and reported dose‐limited bioavailability through a power function [28], resulting in a 53% reduction in *F* at 400 mg versus 100 mg (Figure S2). This aligned with the categorical effect observed in our study, supporting the use of the published power function. Implementation of this function led to a modest improvement in model fit (∆OFV = −4.4). All PK parameters were reestimated after implementing the function, with coefficients fixed to published values (0.541 and 34.7 mg). Reestimated CL/F and V/F parameters decreased 1.8‐fold, indicating that saturation of absorption (*F* < 1) was already evident at 100 mg (Table S3).

IOV in absorption parameters was significant for both *F* (∆OFV = −283.3) and K_A_ (∆OFV = −21.4). IIV was incorporated for all estimated PK parameters during model building, but removed from V_OXF‐SO2_ in the final model, for which IIV was < 10% and excluding IIV had minimal impact on overall model fit (∆OFV = 0.03).

Parameter estimates for the final model are provided in Table 1. Bootstrapping indicated a robust PK model with moderate to high precision (RSE < 32% for all PK parameters) except IIV on *F*. Notably, variability in *F* was high (72.7% coefficient of variation, CV), largely driven by one subject in the 400 mg single dose cohort with markedly reduced plasma exposure. Excluding this subject reduced IIV on *F* to 32.8% and improved precision of the estimate (RSE 53.5% vs. 27.6%; Table S4). However, exclusion had minimal impact (< 15%) on other PK parameter estimates, and this subject was therefore retained in the final model.

**TABLE 1:** Parameter estimates of the final population PK model of oxfendazole, fenbendazole, and oxfendazole sulfone.

	Parameter	Population estimate^a^ (%RSE)^b^	Bootstrapping 95% CI^b^	IIV/IOV, %CV^a^ (%RSE)^b^	Bootstrapping 95% CI^b^
*F*	Relative oral bioavailability	1 *fixed*	—	72.7 (53.5)/ 38.9 (13.8)	17.6–152.3/ 27.6–49.0
K_A_ (h^−1^)	Absorption rate constant	0.695 (9.1)	0.574–0.825	31.1 (26.9)/ 54.1 (25.5)	13.6–51.7/ 20.6–78.4
f_FP_	Fraction of first‐pass metabolism	0.055 (14.3)	0.039–0.070	44.2 (30.9)	23.4–82.4
CL_OXF_/F (L/h)	Apparent clearance oxfendazole	3.54 (17.7)	2.65–5.18	12.7 (31.7)	4.9–20.4
V_OXF_/F (L)	Apparent volume of distribution oxfendazole	67.2 (18.1)	49.2–99.4	19.9 (25.6)	9.6–30.2
CL_FEN_/F (L/h)	Apparent clearance fenbendazole	94.4 (19.2)	68.0–140	92.5 (20.5)	57.5–128
V_FEN_/F (L)	Apparent volume of distribution fenbendazole	3370 (18.1)	2490–4868	87.5 (21.5)	52.9–125
CL_OXF‐SO2_/F (L/h)	Apparent clearance oxfendazole sulfone	15.8 (16.0)	12.4–22.3	23.8 (15.2)	16.7–30.3
V_OXF‐SO2_/F (L)	Apparent volume of distribution oxfendazole sulfone	206 (14.2)	161–282	—	—
f_M_	Fraction of oxfendazole clearance attributed to formation of fenbendazole and oxfendazole sulfone	0.5 *fixed*	—	—	—
*σ*_OXF_	Oxfendazole residual error as SD^c^	0.152 (9.1)	0.127–0.182	—	—
*σ*_FEN_	Fenbendazole residual error as SD^c^	0.421 (10.1)	0.337–0.503	—	—
*σ*_OXF‐SO2_	Oxfendazole sulfone residual error as SD^c^	0.137 (7.4)	0.119–0.158	—	—
*F* = (Dose[mg]/34.7 mg)^−0.541^	Dose effect on *F*	−0.541 *fixed* 34.7 *fixed*	—	—	—

*Note:* Population estimates are given for an adult weighting 70 kg.

^a^ Population mean parameter estimates and IIV calculated by NONMEM. The coefficient of variation (% CV) for the IIV and IOV was calculated from the variance of ETA (ω^2^) as $100\times \sqrt{e^{\omega^{2}}-1}$, except for f_FP_ (fraction of first‐pass metabolism), which was modeled on the logit scale. For f_FP_, CV% on the linear scale was computed from numerical simulation (see Code S2 in Data S1). The original NONMEM variance on the logit scale is provided in Code S1 in Data S1 for reproducibility.

^b^ Precision of parameter estimates, based on nonparametric bootstrap diagnostics (638 successful runs out of 1000) of the final PK model. RSEs (%) are calculated as $100\times \frac{\text{standard deviation}}{\text{mean value}}$. The 95% CIs are based on the 2.5th −97.5th percentiles of the bootstrap parameter estimates.

^c^ Standard deviation (SD) calculated as $\surd \omega^{2}$. Residual error was modeled as additive on the log scale; therefore, the reported SD values are approximately equivalent to a proportional error on the linear scale.

**FIGURE 3:**
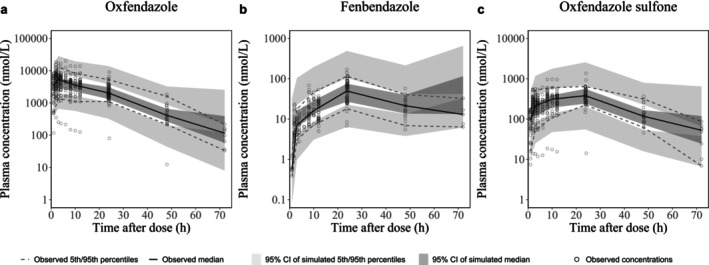
Visual predictive checks for oxfendazole and metabolites. Prediction‐corrected visual predictive checks of the final population PK model for (a) oxfendazole, (b) fenbendazole, and (c) oxfendazole sulfone.

The final model accurately described observed concentration–time profiles without major model misspecifications, as shown in GOF plots (Figure S3) and prediction‐corrected VPCs (Figure 3), although one subject with low bioavailability was evident. An overlay of observed and individually predicted PK profiles is provided in Figure S4.

Eta‐shrinkage was < 25% for all PK parameters except for K_A_ (43.7%). Epsilon shrinkage was < 15% for oxfendazole, fenbendazole, and oxfendazole sulfone. Overall, model diagnostics supported the use of the final model for simulation‐based dose selection.

Secondary PK parameter estimates for oxfendazole and its metabolites are presented in Table 2. Oxfendazole demonstrated slow absorption with a median time to maximum concentration (*T*_MAX_) ranging between 3.5 and 3.7 h across cohorts. After single‐dose administration, oxfendazole plasma exposure increased with dose, exhibiting less than dose‐proportional behavior. A 4‐fold dose increase (cohort 1 vs. 2) resulted in a 2.6‐fold increase in median *C*_MAX_ and a 2.8‐fold increase in AUC_∞_ for oxfendazole, which is reflected by lower dose‐normalized exposure estimates at the higher dose. Less than dose‐normalized behavior was also observed for fenbendazole and oxfendazole sulfone. Plasma concentrations for both metabolites were much lower compared to the parent compound, with fenbendazole showing the lowest exposure. For example, the AUC_∞_ for oxfendazole in cohort 1 was 44‐fold and 9‐fold higher compared to fenbendazole and oxfendazole sulfone, respectively.

**TABLE 2:** Secondary PK parameters for oxfendazole and its metabolites fenbendazole and oxfendazole sulfone.

Secondary PK parameter	Cohort 1 (100 mg, single dose)	Cohort 2 (400 mg, single dose)	Cohort 3 (400 mg, 5 days once daily)
**Oxfendazole**			
AUC_∞_ (ng × h/mL)	17,100 (7960–47,700)	48,000 (7150–104,000)	182,000 (125,000–281,000)
AUC_∞_/Dose ((ng × h/mL)/mg)^a^	171 (79.6–477)	120 (17.9–260)	90.8 (62.7–140)
*C*_MAX_ (ng/mL)	796 (319–1930)	2060 (240–4610)	2790 (1970–4640)
*C*_MAX_/dose ((ng/mL)/mg)^b^	7.96 (3.19–19.3)	5.15 (0.60–11.5)	6.97 (4.93–11.6)
*T*_MAX_ (h)	3.68 (2.52–6.22)	3.57 (2.60–7.69)	3.55 (2.93–4.40)
Terminal half‐life, *t*_½_ (h)	12.3 (10.2–16.2)	13.5 (9.89–17.0)	11.7 (8.94–14.1)
Time above target (days)^c^	1.16 (0.53–2.14)	2.02 (0.49–2.63)	5.74 (5.49–6.15)
**Fenbendazole**			
AUC_∞_ (ng × h/mL)	386 (308–562)	806 (151–1580)	1790 (1410–4600)
AUC_∞_/dose ((ng × h/mL)/mg)^a^	3.86 (3.08–5.62)	2.02 (0.38–3.95)	0.89 (0.71–2.30)
*C*_MAX_ (ng/mL)	5.98 (3.42–7.00)	9.94 (2.55–18.7)	16.1 (11.6–50.5)
*C*_MAX_/dose ((ng/mL)/mg)^b^	0.06 (0.03–0.07)	0.02 (0.01–0.05)	0.04 (0.03–0.13)
*T*_MAX_ (h)	30.0 (20.3–32.8)	26.7 (17.8–38.2)	13.5 (10.7–16.8)
Terminal half‐life, *t*_½_ (h)	31.1 (11.9–50.3)	26.5 (9.39–48.5)	16.0 (8.86–42.2)
**Oxfendazole sulfone**			
AUC_∞_ (ng × h/mL)	1970 (1640 – 5400)	5970 (1160–12,300)	21,940 (16,700–32,500)
AUC_∞_/dose ((ng × h/mL)/mg)^a^	19.7 (16.4–54.0)	14.9 (2.91–30.8)	11.0 (8.37–16.2)
*C*_MAX_ (ng/mL)	50.6 (31.8–122)	142 (22.0–299)	267 (160–348)
*C*_MAX_/dose ((ng/mL)/mg)^b^	0.51 (0.32–1.22)	0.35 (0.06–0.75)	0.67 (0.40–0.87)
*T*_MAX_ (h)	13.0 (11.3–19.8)	16.3 (11.2–21.5)	8.15 (4.80–12.4)
Terminal half‐life, *t*_½_ (h)	8.05 (7.07–12.2)	9.15 (6.86–11.9)	8.35 (6.22–11.1)

*Note:* All values are given as median (5th to 95th percentile).

^a^ Normalized by the total dose (100, 400, and 2000 mg in cohorts 1, 2, and 3, respectively).

^b^ Normalized by the daily dose.

^c^ Time above a target concentration of 200 ng/mL in plasma.

After 5 days of dosing, higher *C*_MAX_ values were observed for oxfendazole, fenbendazole, and oxfendazole sulfone compared to the 400 mg single‐dose cohort, indicating minor accumulation of the parent and more pronounced accumulation of the metabolites (noting that these were different participants with varying body weights). Oxfendazole reached the target concentration of 200 ng/mL in all cohorts, with longer durations above the target concentration at higher doses and after multiple doses. Oxfendazole and oxfendazole sulfone had an elimination half‐life of approximately 12 and 8 h, respectively, which was consistent across dosing regimens. Fenbendazole showed a longer half‐life and high variability (wide 95% CIs) in this parameter, mainly due to high IIV in clearance estimates for fenbendazole.

### 3.4 Simulation of Different Dosing Scenarios

**FIGURE 4:**
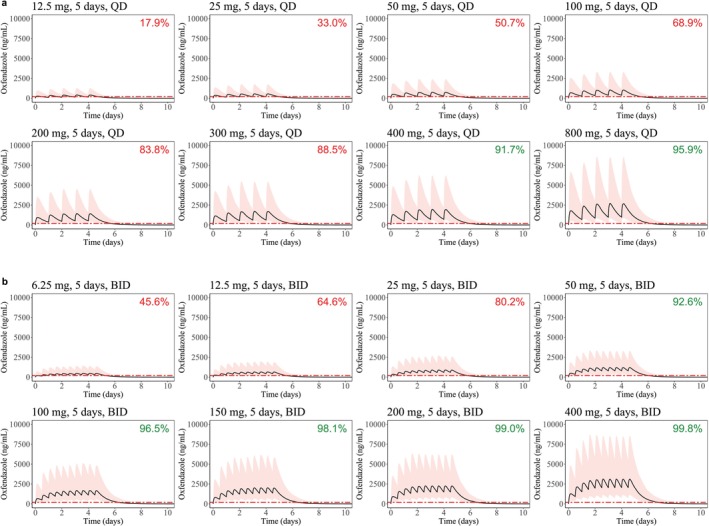
Simulated PK profiles for oxfendazole and probability of target attainment. Plasma concentration–time profiles for different dosing regimens were simulated using the final population PK model for a 60 kg adult. (a) Once daily dosing (QD) for 5 days with 12.5–800 mg oxfendazole; (b) twice daily dosing (BID) for 5 days with 6.25–400 mg oxfendazole per dosing occasion. Black solid lines represent the median of the simulated oxfendazole plasma concentrations over time, with the 90% prediction interval shown as shaded area (5th and 95th percentiles). The horizontal red line represents the putative target concentration (C_TARGET_ = 200 ng/mL). Percentages in the upper right corner indicate PTAs (defined as the percentage of patients with a minimum of 5 days total time above the target concentration of 200 ng/mL).

The final population PK model was applied to evaluate various dosing regimens for oxfendazole in adults (60 kg body weight). The aim was to achieve an oxfendazole plasma concentration of 200 ng/mL over a minimum duration of 5 days and for at least 90% of the population. Sixteen dosing regimens were explored, spanning different daily doses (12.5–800 mg) and dosing frequencies (once or twice daily dosing) over a 5‐day period. Simulated oxfendazole plasma concentration–time profiles for the various dosing regimens are shown in Figure 4, along with corresponding PTAs. Key exposure metrics from the simulated oxfendazole PK profiles are summarized in Figure S5.

Simulations revealed substantial variability in oxfendazole plasma exposure, largely driven by high variability in absorption. Splitting a total daily dose into two administrations (12 h apart) led to accumulation over time and increased overall exposure (AUC_∞_, *C*_MAX_, and times above the target concentration; see Discussion for rationale). The simulations predicted that once daily dosing with 400 mg oxfendazole for 5 days is required to reach therapeutic target exposures (> 90% PTA). However, a lower total daily dose of 100 mg oxfendazole, split into two daily administrations (as 50 mg twice daily), also achieved the target exposure level. A further increase in maintenance doses resulted in only a minor increase in the time above the target concentration, but led to higher AUC_∞_ and *C*_MAX_ levels.

The median simulated *C*_MAX_ for once daily 400 mg oxfendazole for 5 days and twice daily 50 mg oxfendazole for 5 days was 2528 ng/mL (SD ±2456; 90% CI: 865–8314 ng/mL) and 1376 ng/mL (SD ±1285; 90% CI: 929–7264 ng/mL), respectively (Figure S5b).

### 3.5 Sensitivity Analysis (Body Weight)

To further evaluate the proposed dosing regimens, additional simulations were performed across body weights ranging from 42 to 75 kg, representative of the lower to upper body‐weight extremes of the study population (Figure S6). Simulations focused on the primary target concentration of 200 ng/mL. There was a trend towards lower PTA at higher body weight across all dosing regimens, reflecting lower systemic exposure as body weight increased. For the proposed regimens of 400 mg once daily or 50 mg twice daily administered for 5 days, PTA remained above 90% at lower body weights and declined modestly with increasing body weight. At the upper body‐weight extreme of 75 kg, PTA remained above 85% for both regimens. Corresponding simulated exposure metrics at lower and upper body‐weight extremes are provided in Figure S7.

### 3.6 Sensitivity Analysis (Target Concentration)

**FIGURE 5:**
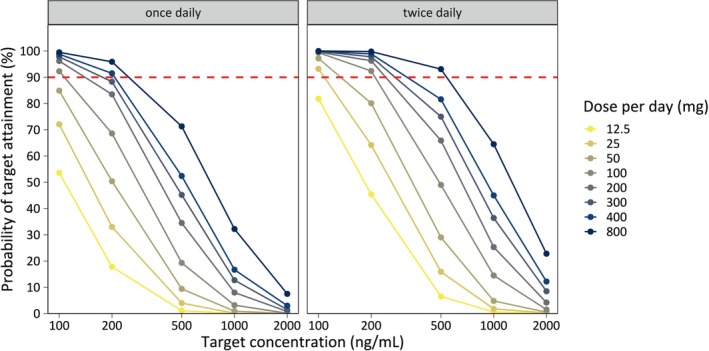
Probabilities of target attainment for different target concentrations. PTAs are shown for various target concentrations (100–2000 ng/mL) and dosing regimens, namely 5 days of dosing with 12.5–800 mg daily doses of oxfendazole, given either as a single daily dose (left panel) or split into two daily doses (right panel). Simulations were performed for 1000 adults per dosing regimen assuming a body weight of 60 kg. PTA is the percentage of simulated subjects achieving plasma oxfendazole concentrations above target concentrations for a minimum duration of 5 days.

Given the uncertainty around the exposure target, a sensitivity analysis was conducted for a range of target concentrations (100–2000 ng/mL oxfendazole) for a 60‐kg adult. The median simulated concentration–time profiles of oxfendazole in human plasma, relative to the different target concentrations, are shown in Figure S8 (dosing regimens as in Figure 4), along with distributions of simulated times above the target concentration (Figure S9). Corresponding PTAs across target concentrations and dosing regimens are shown in Figure 5.

At a target concentration of 100 ng/mL, once daily dosing with 100 mg oxfendazole—or alternatively twice daily dosing with 12.5 mg oxfendazole (25 mg daily dose)—for 5 days achieved > 90% PTA. However, at a target concentration of 200, 500, 1000, and 2000 ng/mL, the PTA with 100 mg oxfendazole given once daily was only 68.6%, 19.3%, 3.2%, and 0.1%, respectively. A once daily dose of 400 mg oxfendazole or 50 mg twice daily for 5 days was required to achieve > 90% PTA at 200 ng/mL.

For target concentrations ≥ 500 ng/mL, none of the investigated doses achieved the exposure target with once daily dosing, while only the highest dose (400 mg twice daily) reached the exposure target. All dose levels—whether once or twice daily—failed to achieve a 90% PTA at target concentrations ≥ 1000 ng/mL.

## 4 Discussion

Global efforts to eliminate onchocerciasis face a critical challenge due to the lack of a macrofilaricidal drug capable of eradicating adult parasites. To achieve the ambitious 2030 elimination goals [5], alternative strategies beyond long‐term MDA with ivermectin are needed. A macrofilaricidal drug could shorten treatment durations, improve individual clinical care, and support “mop‐up” campaigns to clear residual infections [30, 31].

Oxfendazole, a promising macrofilaricidal veterinary drug [14, 21, 23], is currently under clinical development for helminth infections. A first‐in‐human single ascending dose (SAD) and multiple ascending dose (MAD) Phase 1 study in healthy, primarily Caucasian volunteers evaluated an oral solution and demonstrated favorable pharmacokinetics and a good safety profile [25, 26, 28, 32]. More recently, an immediate‐release tablet formulation was developed to facilitate use in endemic settings and support broader application in the target population. A Phase 1 bioavailability study in healthy African adults evaluated this tablet formulation, confirming its safety, tolerability, and PK (based on NCA analysis, results reported separately) [27].

This study builds on earlier Phase 1 trials of oxfendazole by providing the first population PK analysis of a field‐applicable tablet formulation in African adults. The developed model adequately described the pharmacokinetics of oxfendazole and its major metabolites and was used to evaluate potential dosing regimens for future clinical trials.

### 4.1 Pharmacokinetic Properties of the Oxfendazole Tablet Formulation

Oxfendazole plasma concentration–time profiles were effectively described by a first‐order absorption model with first‐pass metabolism to oxfendazole sulfone, followed by a one‐compartment disposition model with linear elimination. This structural model aligns with findings from prior SAD and MAD studies in healthy, primarily Caucasian volunteers [28, 32].

In our study, absorption of oxfendazole from an immediate‐release tablet formulation was relatively slow (median *T*_MAX_ of 3.5–3.7 h). In contrast, the prior SAD study reported faster absorption from an oral suspension (median *T*_MAX_~2 h) [26, 28]. In both studies, oxfendazole was administered in a fasted state, minimizing the impact of drug–food interactions. The delayed absorption in our study is likely attributed to slower dissolution of the tablet in gastrointestinal fluids compared to a suspension [25, 28]. Notably, oxfendazole is a biopharmaceutics classification system (BCS) class II compound due to its low aqueous solubility and high permeability, supporting this interpretation.

Regarding the extent of absorption, we observed a less than dose‐proportional increase in *C*_MAX_ and AUC_∞_, as reported previously [26, 28]. This nonlinearity is likely due to its incomplete dissolution in gastrointestinal fluids, resulting in reduced bioavailability at higher doses. In our final model, we incorporated a previously established dose‐dependent absorption function to simulate dosing regimens beyond the two categories explored in our study. Whether using a categorical function or the previously published power model, we observed consistent effect sizes affirming the robustness of our approach.

Our study revealed moderate IIV in K_A_. A substantial portion of the variability in K_A_ could be attributed to IOV, indicating random fluctuations in absorption rate within the same individual across different dosing occasions. We also identified substantial variability in relative bioavailability (IIV 72.7%), largely driven by one participant in the 400 mg/single‐dose cohort exhibiting markedly low plasma exposure. Excluding this participant reduced the IIV estimate for bioavailability, but variability remained substantial. Variations in gastric motility, luminal pH, and transporter expression (oxfendazole is a BCRP substrate [33]) may all contribute to this variability.

Strict comparison of absorption parameters between the tablet formulation in the present study and the oral suspension in prior studies [25, 26, 28, 32] is limited, as each formulation was evaluated in a separate study population. Disparities in absorption rate and extent may reflect formulation effects and demographic differences.

The disposition of oxfendazole was well captured by a one‐compartment model, with an estimated apparent clearance (CL_OXF_/F) and volume of distribution (V_OXF_/F) of 3.54 L/h and 67.2 L, respectively. These results indicate that oxfendazole is a low extraction ratio drug with moderate distribution, as previously described [28]. Differences in CL_OXF_/F and V_OXF_/F compared to the prior FIH study can partially be attributed to reduced bioavailability in our study relative to the reference dose in the FIH study (Figure S1).

The elimination half‐life of oxfendazole was ~12 h and consistent across cohorts. Slightly shorter half‐lives were reported in prior Phase 1 studies (SAD: 8.50–11.0 h [25, 28]; MAD: 9.21–11.8 h [32]), though differences were small. Minimal accumulation of oxfendazole was observed after 5 days of once daily dosing, consistent with prior findings.

Oxfendazole was the primary moiety in human plasma, with substantially lower exposure observed for its metabolites. The main metabolite was oxfendazole sulfone, and minimal metabolism to fenbendazole was found, consistent with previous findings [25, 26]. The prior SAD study likewise reported low‐grade metabolism along with low renal clearance, suggesting oxfendazole is primarily excreted through biliary pathways [25]. However, a mass balance study has not been conducted, leaving the fraction of oxfendazole that actually undergoes metabolism unknown. To address this structural unidentifiability, we assumed that 50% of oxfendazole clearance each was attributed to its conversion to fenbendazole and oxfendazole sulfone. Consequently, PK parameters for the metabolites are scaled relative to the unknown true fraction metabolized, rather than representing absolute physiological values [34].

The exposure metrics derived from the population PK model were generally consistent with the descriptive PK parameters from the NCA analysis in the same study population (reported separately [27]), with similar findings for dose‐limited absorption, delayed *T*_MAX_ compared to the oral solution, minor accumulation of oxfendazole after once‐daily dosing, and lower metabolite exposure relative to the parent compound. Some differences in metabolite parameters likely reflect methodological differences between model‐based and noncompartmental approaches. In the NCA analysis, AUC_∞_ and *t*_1/2_ for metabolites could not be estimated in all cohorts. A potential sample mix‐up affecting a single 6‐h timepoint in two participants was corrected in the population PK analysis. This correction reduced IIV on K_A_ (31% vs. 45% CV) and slightly reduced residual error, without meaningful impact on other model parameters or interpretation. A key strength of this study is the use of nonlinear mixed‐effects modeling, which allowed integration of data across dosing cohorts and enabled simulation‐based evaluation of dosing regimens.

### 4.2 Dose Finding Studies

Drug discovery for onchocerciasis heavily relies on surrogate parasite models of human filarial disease, as the full life cycle of *O. volvulus* occurs only in humans and nonhuman primates [35]. The in vivo efficacy of oxfendazole against rodent filarial nematodes has been evaluated in the *L. sigmodontis* mice model, demonstrating excellent macrofilaricidal activity [21]. Sterile cure was achieved with a 12.5 mg/kg oral dose administered twice daily for 5 days, or a 25 mg/kg subcutaneous dose once daily for the same duration. These findings indicated time‐dependent efficacy of oxfendazole, achieved by maximizing time above a minimum inhibitory concentration (~100 ng/mL).

Based on these preclinical targets, we defined a conservative human plasma target as a minimum of 5 days above 200 ng/mL oxfendazole. Simulations predicted that a daily dose of 400 mg oxfendazole once daily (or 50 mg twice daily) is required to achieve a PTA greater than 90% in a 60‐kg adult. When extending the simulations across the adult body‐weight range (42–75 kg), PTA showed a moderate decrease with increasing body weight, reflecting lower systemic exposure at higher body weight. However, even at the upper body‐weight extreme of 75 kg, PTA remained above 85% for the proposed regimens. These findings suggest that the proposed dosing regimens maintain high probabilities of target attainment across a clinically relevant adult body‐weight range, although body‐weight–based dose adjustments could be explored in future clinical studies.

The lower total daily dose required for the twice‐daily regimen can be explained by several factors. Splitting the daily dose into two administrations (12 h apart) reduces the impact of dose‐limited absorption, thereby improving relative bioavailability and, consequently, systemic exposure. Additionally, twice daily dosing leads to drug accumulation, resulting in higher exposure and prolonged time above the target concentration.

Importantly, the predicted effective dosing regimens are associated with a low risk of safety and tolerability concerns. Previous Phase 1 clinical trials demonstrated that oxfendazole, administered as an oral suspension, was well tolerated at single doses up to 60 mg/kg (equivalent to 3600 mg for a 60 kg subject), and multiple daily doses of up to 15 mg/kg (equivalent to 900 mg for a 60 kg subject) over 5 days, with no higher doses tested [25, 28]. Although a few nonserious adverse events (AEs) were reported, there was no dose‐dependent increase in AEs, and no serious AEs or deaths occurred. Exposure levels in these studies were substantially higher than the simulated exposures for the predicted effective dosing regimens in our study, supporting an acceptable safety margin.

Nevertheless, caution is warranted since potential toxicity similar to flubendazole cannot be excluded [36]. Both compounds belong to the benzimidazole class. The development of flubendazole, identified as a potent teratogen with an unfavorable risk/benefit ratio, was discontinued for onchocerciasis [37]. Oxfendazole also showed toxicity in specific organs, such as testicular toxicity in rats [23, 37], warranting precautionary measures like contraception and emphasizing the need for ongoing vigilance in clinical trials.

Finally, several limitations of the present analysis should be acknowledged. Translation of preclinical efficacy targets derived from surrogate parasites of *O. volvulus* to human onchocerciasis remains inherently uncertain. While the *L. sigmodontis* mouse model is a well‐established tool in antifilarial drug development, differences in infection site, disease pathology, host–parasite interactions, parasite biology, and PK patterns between mice and men pose important translational challenges [8, 38]. To address uncertainty around exposure targets, we conducted a sensitivity analysis across a wide range of target concentrations, revealing a high sensitivity of target attainment to the selected target value.

In addition, uncertainty in PTA predictions arises from uncertainty in the underlying PK parameter estimates, as reflected by their relative standard errors. As highlighted by Colin et al. [39], interpretation of PTA simulations should ideally account for this parameter uncertainty by estimating CIs around PTA curves. In the present study, PTA analyses should therefore be regarded as exploratory and hypothesis‐generating under plausible assumptions. Specifically, simulations were based on PK parameter estimates from the final population PK model in healthy volunteers and a provisional exposure target derived from preclinical studies. The analysis supports the identification of plausible dosing regimens for further clinical evaluation rather than clinical decision‐making. Future studies conducted in onchocerciasis patients should refine target attainment analyses using PK parameters estimated in the patient population, following confirmation of clinically relevant exposure targets. At that stage, probabilistic approaches that explicitly propagate parameter uncertainty and provide CIs around PTA curves will be important to support robust dose optimization and informed clinical decision‐making.

Ultimately, prospective clinical trials remain essential to confirm macrofilaricidal efficacy against adult *O. volvulus*.

## 5 Conclusions

The developed population pharmacokinetic model adequately described oxfendazole pharmacokinetics in healthy African adults receiving a field‐applicable tablet formulation, demonstrating substantial variability in exposure and confirming dose‐limited absorption. PK modeling and simulation predicted that a 400 mg once daily or 50 mg twice daily dose of oxfendazole over 5 days achieves exposure targets (≥ 200 ng/mL for ≥ 5 days) with no safety concerns. Our findings support oxfendazole's potential as a macrofilaricidal drug candidate for onchocerciasis and provide a valuable tool to guide dosing optimization for continued clinical development.

## Author Contributions

F.A. wrote the manuscript. J.K., E.A., I.S., F.A., R.M.H., and J.T. designed the research. F.A., A.A., R.M.H., G.N., H.M., S.J., E.A., E.R., J.K., F.B.D.S.R., S.S., I.S., and J.T. performed the research. F.A., A.A., R.M.H., and J.T. analyzed the data.

## Funding

This project has received funding from the European Union's Horizon 2020 research and innovation program (grant agreement no. 815628—HELP). This research was also partly funded by the Wellcome Trust (grant number 220211, London, UK) awarded to Richard Hoglund and Joel Tarning. For the purpose of open access, the author has applied a CC BY public copyright license to any Author Accepted Manuscript version arising from this submission.

## Conflicts of Interest

The authors declare no conflicts of interest.

## Supporting information


**Data S1:** psp470189‐sup‐0001‐DataS1.docx.
