# Supplementary material for: Repurposing Oxfendazole for Onchocerciasis: Population Pharmacokinetics of a Tablet Formulation in Healthy African Adults
Source: CPT Pharmacometrics Syst Pharmacol. 2026 Feb 9;15(2):e70189. doi: 10.1002/psp4.70189 (PMC12896373; doi:10.1002/psp4.70189)
Supplement: Supplementary file 1 — Data S1: psp470189‐sup‐0001‐DataS1.docx. [file PSP4-15-e70189-s001.docx]

**Supplementary Information**

**Repurposing oxfendazole for onchocerciasis: Population pharmacokinetics of a tablet formulation in healthy African adults**

Frauke Assmus, Ayorinde Adehin, Richard M. Hoglund, Gloria Nyaulingo, Hussein Mbarak, Said Jongo, Eveline Ackermann, Elisabeth Reus, Jennifer Keiser, Fabiana Barreira Da Silva Rocha, Sabine Specht, Ivan Scandale, Joel Tarning

**Content**

**Table S1.** Summary of HPLC-MS/MS conditions.

**Table S2.** Baseline demographic data for the PK analysis population.

**Table S3.** Comparison of parameter estimates of the population PK model following implementation of the dose effect on F.

**Table S4.** Sensitivity analysis for parameter estimates of the final population PK model.

**Figure S1.** PK profiles of subjects with potential sample mix-up.

**Figure S2.** Dose effect on relative bioavailability for oxfendazole as described previously.

**Figure S3.** Goodness-of-fit of the final population PK model for oxfendazole and its metabolites fenbendazole and oxfendazole sulfone.

**Figure S4.** Observed and individually predicted PK profiles.**Figure S5.** Simulated oxfendazole plasma exposure for different dosing regimens.

**Figure S6.** Probability of target attainment across body weights at a target concentration of 200 ng/mL.

**Figure S7.** Simulated oxfendazole plasma exposure stratified by body weight for selected dosing regimens.

**Figure S8.** Simulated oxfendazole PK profiles for once-daily and twice-daily dosing regimens across target concentrations.

**Figure S9.** Simulated oxfendazole time above target concentration for once-daily and twice-daily dosing regimens across target concentrations.

**Code S1.** NONMEM code of the final population PK model.

**Code S2.** R code for the computation of CV% for fraction of first-pass metabolism.

**Table S1.** Summary of HPLC-MS/MS conditions

| **Parameter** | **Details** |
| --- | --- |
| **Analyte** | - Oxfendazole - Fenbendazole - Oxfendazole sulfone |
| **Sample preparation** | - Plasma was precipitated with internal standards - The mixture was vortex-mixed and centrifuged - 5 µL of the supernatant injected for drug quantification |
| **Instrumentation** | - Agilent 1200 HPLC pump (Agilent Technologies Inc, Santa Clara, CA, USA) - CTC PAL Autosampler (CTC Analytics AG, Zwingen, Switzerland) - HotDog 5090 column oven, Prolab GmbH, Reinach, Switzerland - TSQ Quantum Access mass spectrometer (Thermo Fisher Scientific, San Jose, CA, USA) |
| **LC column** | - Hypersil Gold, 2.1 x 50 mm, 3 µm, Thermo Fisher Scientific Inc., Waltham, MA, USA - Column Temperature: 40 °C |
| **Mobile phase and gradient** | - Mobile phase A: 0.5% formic acid in water - Mobile phase B: 0.2% formic acid in methanol - 0–0.2 min: 95% A - 1.5–3.55 min: 2% A - 3.6–4.0 min: 95% A - Flow rate: of 0.5 mL/min |
| **Inter-assay precision (% CV)** | - 3.4% – 15.2% |
| **Inter-assay accuracy (%)** | - 90.5% – 105.1% |
| **Recovery** | - 95.2% – 105.1% |
| **Lower limit of quantification** | - Oxfendazole: 2 ng/mL - Fenbendazole: 1 ng/mL - Oxfendazole sulfone: 1 ng/mL |

**Table S2.** Baseline demographic data for the PK analysis population.

| **Characteristic** | **Cohort 1**  **100 mg OXF**  **(n = 8)** | **Cohort 2**  **400 mg OXF**  **(n = 8)** | **Cohort 3**  **400 mg OXF, 5 days**  **(n = 8)** | **All PK data**  **(n=24)** |
| --- | --- | --- | --- | --- |
| **Sex, n (%)**  Female  Male | 3 (37.5)  5 (62.5) | 3 (37.5)  5 (62.5) | 4 (50)  4 (50) | 10 (41.7)  14 (58.3) |
| **Age [years]** | 30  (24 - 38) | 27  (19 - 41) | 25.5  (19 - 33) | 28  (19 - 41) |
| **Weight [kg]** | 57  (49 - 61) | 59.5  (42 - 75) | 58.5  (49 - 74) | 58  (42 - 75) |
| **Height [cm]** | 165.5  (155.0 - 182.5) | 158.5  (150.0 - 170.0) | 162.0  (145.5 - 178.0) | 161.5  (145.5 - 182.5) |
| **BMI [kg/m^2^]** | 20.3  (18.3 - 22.3) | 22.9  (18.4 - 28.9) | 21.5  (18.9 - 28.9) | 21.4  (18.3 - 28.9) |

**All participants were of Black African ethnicity.**

**Abbreviations:** OXF, oxfendazole; BMI, body mass index

All values are given as median (minimum - maximum range), except sex (% of subjects).

**Table S3.** Comparison of parameter estimates of the population PK model following implementation of the dose effect on F.

| **Parameter** | **Literature model^a^:**  **log(F) = -0.541 x log (Dose/34.7mg)** | |  | **This study data: Dose effect as a categorical function on F** | |
| --- | --- | --- | --- | --- | --- |
|  | **Population estimate^b^** | **IIV/IOV, %CV^b^** |  | **Population estimate^b^** | **IIV/IOV, %CV^b^** |
| Relative oral bioavailability, F | 1 *fixed* | 72.7/ 38.9 |  | 1 *fixed* | 72.7/ 38.9 |
| Absorption rate constant, K_A_ (h^-1^) | 0.695 | 31.1 /54.1 |  | 0.695 | 31.1 /54.1 |
| Fraction of first pass metabolism, f_FP_ | 0.055 | 46.0 |  | 0.055 | 42.2 |
| Apparent clearance oxfendazole, CL_OXF_/F (L/h) | 3.54 | 12.7 |  | 6.2 | 12.7 |
| Apparent volume of distribution oxfendazole, V_OXF_/F (L) | 67.2 | 19.9 |  | 118 | 19.9 |
| Apparent clearance fenbendazole, CL_FEN_/F (L/h) | 94.4 | 92.5 |  | 166 | 92.5 |
| Apparent volume of distribution fenbendazole, V_FEN_/F (L) | 3370 | 87.5 |  | 5920 | 87.5 |
| Apparent clearance oxfendazole sulfone, CL_OXF-SO2_/F (L/h) | 15.8 | 23.8 |  | 27.7 | 23.8 |
| Apparent volume of distribution oxfendazole sulfone, V_OXF-SO2_/F (L) | 206 | - |  | 361 | - |
| Fraction of oxfendazole clearance attributed to formation of fenbendazole and oxfendazole sulfone, respectively, f_M_ | 0.5 *fixed* | - |  | 0.5 *fixed* | - |
| Variance of oxfendazole residual error, σ_OXF_ | 0.0231 | - |  | 0.0231 | - |
| Variance of fenbendazole residual error, σ_FEN_ | 0.177 | - |  | 0.177 | - |
| Variance of oxfendazole sulfone residual error, σ_OXF-SO2_ | 0.0189 | - |  | 0.0189 | - |
| Dose effect on F | *-0.541 fixed; 34.7 fixed* | - |  | -53.5 %^c^ | - |

Population estimates are given for an adult weighing 70 kg. The table compares the application of a literature model (log(F) = –0.541 × log(Dose/34.7 mg)) for the dose effect on F versus estimation using a categorical function of dose, as implemented in this study. The difference in the dose effect on F resulting from the use of the literature model versus the categorical function is highlighted in red. ^a^ Bach et al. Population Pharmacokinetic Model of Oxfendazole and Metabolites in Healthy Adults following Single Ascending Doses. AAC. 2021;65(4). ^b^ Population mean parameter estimates, IIV and IOV calculated by NONMEM. CV % for the IIV and IOV was calculated as $100\times\sqrt{e^{\omega^{2}}-1}$, except for f_FP_. For f_FP_, CV% was computed from numerical simulation (**Supplementary Code S2**).  ^c^ Estimated % reduction in F when increasing the dose from 100mg (reference) to 400 mg.

**Table S4.** Sensitivity analysis for parameter estimates of the final population PK model.

| **Parameter** | **All subjects (n=24)** | |  | **Exclusion of one subjects in 400 mg, single dose cohort** | |
| --- | --- | --- | --- | --- | --- |
|  | **Population estimate^a^ (%RSE)^b^** | **IIV/IOV, %CV^a^**  **(%RSE)^b^** |  | **Population estimate^a^ (%RSE)^b^** | **IIV/IOV, %CV^a^**  **(%RSE)^b^** |
| F | 1 *fixed* | 72.7 (53.5) / 38.9 (13.8) |  | 1 *fixed* | 32.8 (27.6) **/** 38.9 (12.2) |
| K_A_ (h^-1^) | 0.695 (9.1) | 31.1 (26.9) / 54.1 (25.5) |  | 0.690 (9.2) | 31.3 (27.8)/ 55.2 (24.6) |
| f_FP_ | 0.055 (14.3) | 42.2 (30.9) |  | 0.056 (14.0) | 43.7 (29.6) |
| CL_OXF_/F (L/h) | 3.54 (17.7) | 12.7 (31.7) |  | 3.14 (9.7) | 11 (39.8) |
| V_OXF_/F (L) | 67.2 (18.1) | 19.9 (25.6) |  | 60.2 (11.9) | 21.2 (24.6) |
| CL_FEN_/F (L/h) | 94.4 (19.2) | 92.5 (20.5) |  | 85.1 (12.7) | 90.6 (20.5) |
| V_FEN_/F (L) | 3370 (18.1) | 87.5 (21.5) |  | 3030 (12.6) | 86.7 (21.6) |
| CL_OXF-SO2_/F (L/h) | 15.8 (16.0) | 23.8 (15.2) |  | 14.1 (7.7) | 22.6 (14.4) |
| V_OXF-SO2_/F (L) | 206 (14.2) | - |  | 188 (8.5) | - |
| f_M_ | 0.5 *fixed* | - |  | 0.5 *fixed* | - |
| σ_OXF_ | 0.0231 (9.0) | - |  | 0.0225 (8.9) |  |
| σ_FEN_ | 0.177 (10.1) | - |  | 0.177 (9.8) | - |
| σ_OXF-SO2_ | 0.0189 (7.4) | - |  | 0.0188 (7.1) | - |
| Dose effect on F | *-0.541 fixed; 34.7 fixed* | - |  | *-0.541 fixed; 34.7 fixed* | - |

Population estimates are given for an adult weighting 70 kg. The impact on the interindividual variability (IIV) of F is highlighted in red.

^a^ Population mean parameter estimates and IIV calculated by NONMEM. The coefficient of variation (% CV) for the IIV and IOV was calculated as $100\times\sqrt{e^{\omega^{2}}-1}$,except for f_FP_. For f_FP_, CV% was computed from numerical simulation (**Supplementary Code S2**).

^b^ Precision of parameter estimates, based on nonparametric bootstrap diagnostics of the final PK model. RSEs (%) are calculated as $100\times\frac{standard deviation}{mean value}$. The 95% CIs are based on the 2.5^th^ –97.5^th^ percentiles of the bootstrap parameter estimates.


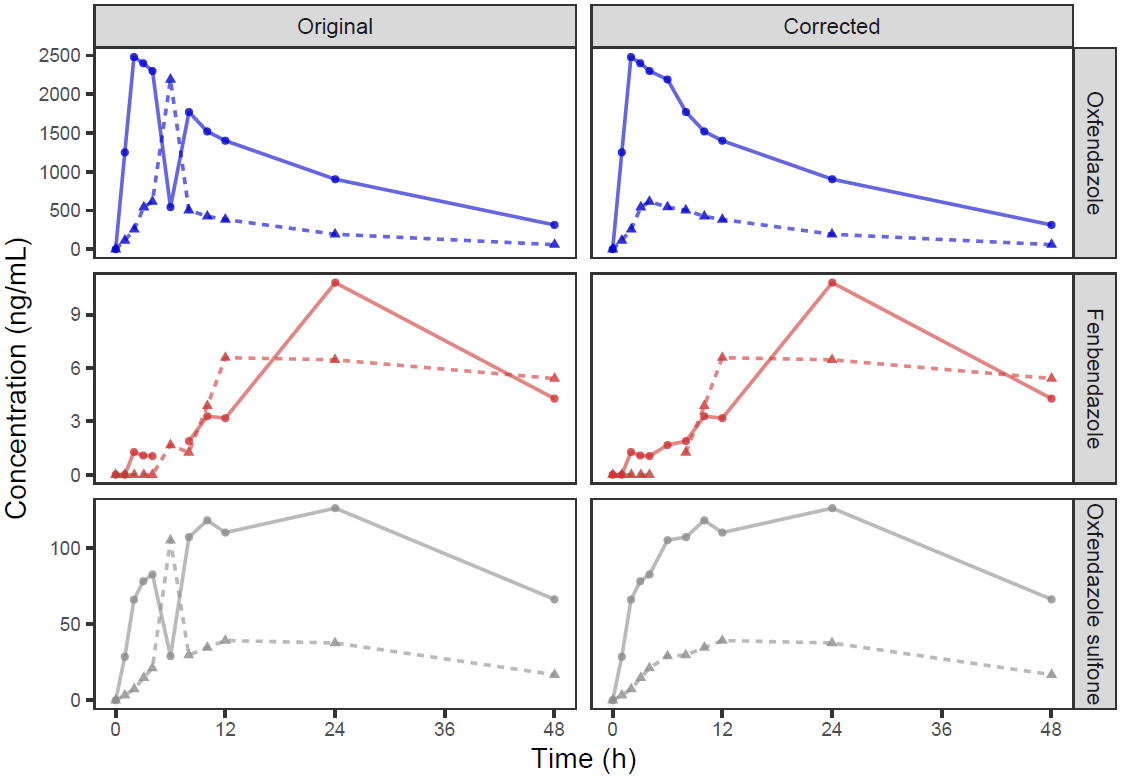


**Fig S1.** **PK profiles of subjects with potential sample mix-up.**

Plasma concentration–time profiles for oxfendazole, fenbendazole, and oxfendazole sulfone following administration of 100 mg oxfendazole (single dose, Cohort 1). Each line represents an individual participant: solid lines correspond to Subject A, and dashed lines to Subject B. Profiles are shown before (left) and after (right) correction of a potential sample mix-up at the 6-hour time point. The correction affected these two participants from Cohort 1.

**Fig S2.** **Dose effect on relative bioavailability for oxfendazole as described previously [1].**

Dose-limited bioavailability (F) was described by a power function, $F={(\frac{Dose (mg)}{34.7mg})}^{-0.541}$, based on a wide range of oxfendazole doses (0.5 to 60 mg/kg) administered as an oral suspension in healthy Caucasian volunteers. The lowest dose in this study was 34.7 mg oxfendazole, providing a reference with F set to 1 (grey dashed line). At 100 mg and 400 mg oxfendazole, F was estimated as 0.56 and 0.27, respectively, corresponding to a 53% reduction in F when comparing these two dose levels (red dashed lines).

**References:**

1. Bach T, Murry DJ, Stebounova LV, Deye G, Winokur P, An G. Population Pharmacokinetic Model of Oxfendazole and Metabolites in Healthy Adults following Single Ascending Doses. Antimicrobial agents and chemotherapy. 2021;65(4).


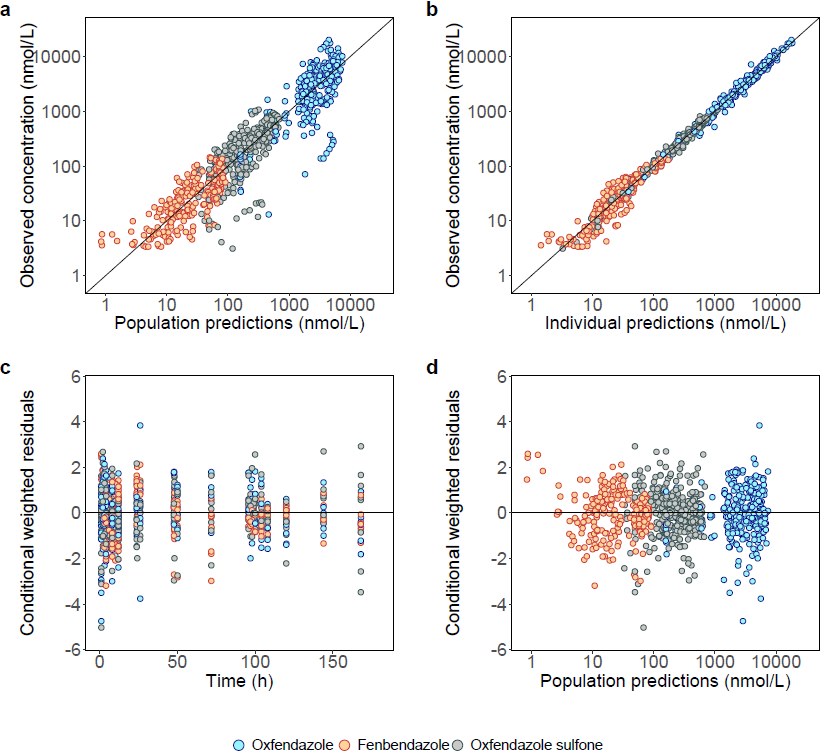


**Fig S3.** **Goodness-of-fit of the final population PK model for oxfendazole and its metabolites fenbendazole and oxfendazole sulfone.**

**a)** observed versus population predicted concentrations, **b)** observed versus individually predicted concentrations, **c)** conditionally weighted residuals versus time, and **d)** conditionally weighted residuals versus population predicted concentrations. Observations are represented by circles. Solid black lines represent the line of identity (**a,b**) or zero line (**c,d).**


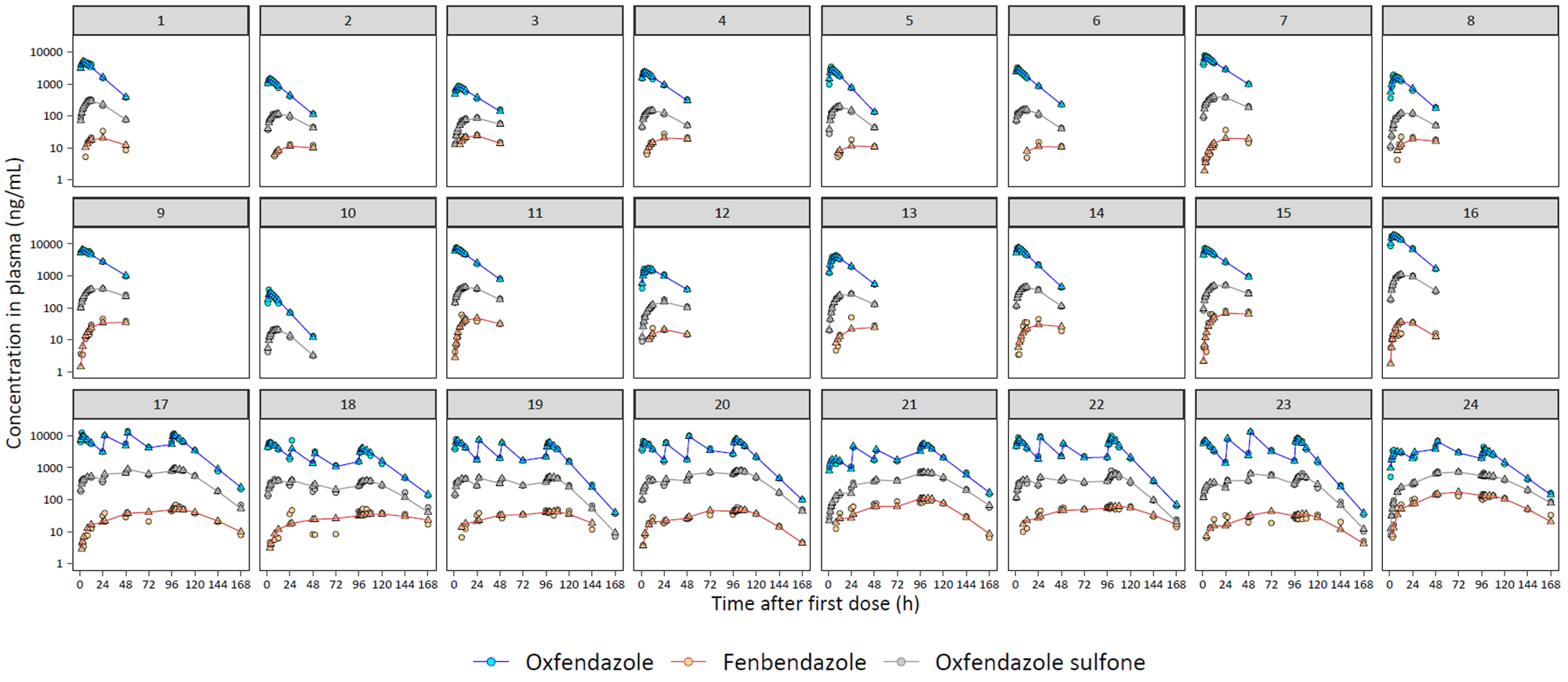


**Fig S4.** **Observed and individually predicted PK profiles.**

Overlay of observed (triangles) and individually predicted (circles) plasma concentration-time profiles for oxfendazole (blue), fenbendazole (red) and oxfendazole sulfone (grey). PK profiles are shown for the 24 subjects included in the final population PK analysis, stratified by cohort: 100 mg/kg single dose (upper panel); 400 mg single dose (middle panel), and 400 mg once daily for 5 days (lower panel).


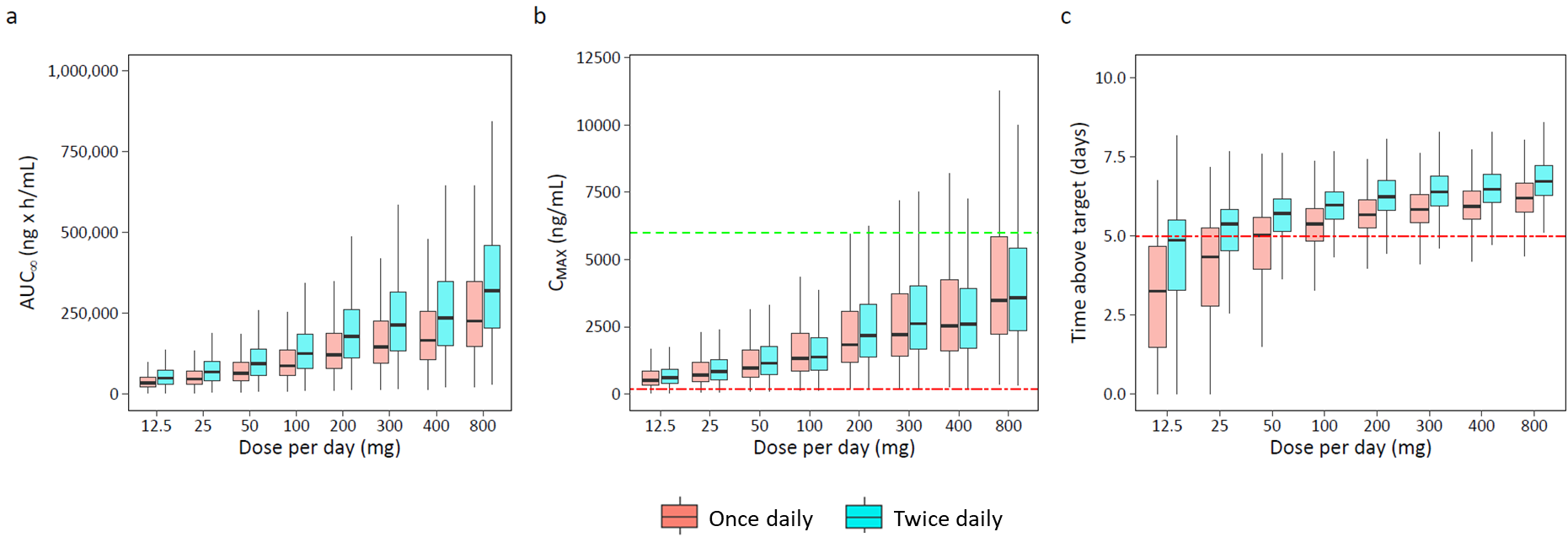


**Fig S5.** **Simulated oxfendazole plasma exposure for different dosing regimens.**

Comparison of once daily dosing versus splitting the daily dose into two administrations (total daily dose 12.5 to 800 mg oxfendazole for 5 days). Boxes represent the interquartile range (IQR), bold lines the median, whiskers 1.5×IQR. **a)** AUC_∞_, **b)** C_MAX_. **c)** Time above the target concentration. In panel b, the horizontal red line marks the putative target concentration (C_TARGET_ = 200ng/mL); the horizontal green line shows the median C_MAX_ from a previous MAD study with 15 mg/kg oxfendazole [1], representing a well-tolerated level. In panel c, the horizontal red line represents the minimum target duration above C_TARGET_ (5 days).

**References:**

1 Bach T, Galbiati S, Kennedy JK, Deye G, Nomicos EYH, Codd EE, et al. Pharmacokinetics, Safety, and Tolerability of Oxfendazole in Healthy Adults in an Open-Label Phase 1 Multiple Ascending Dose and Food Effect Study. Antimicrobial agents and chemotherapy. 2020;64(11).


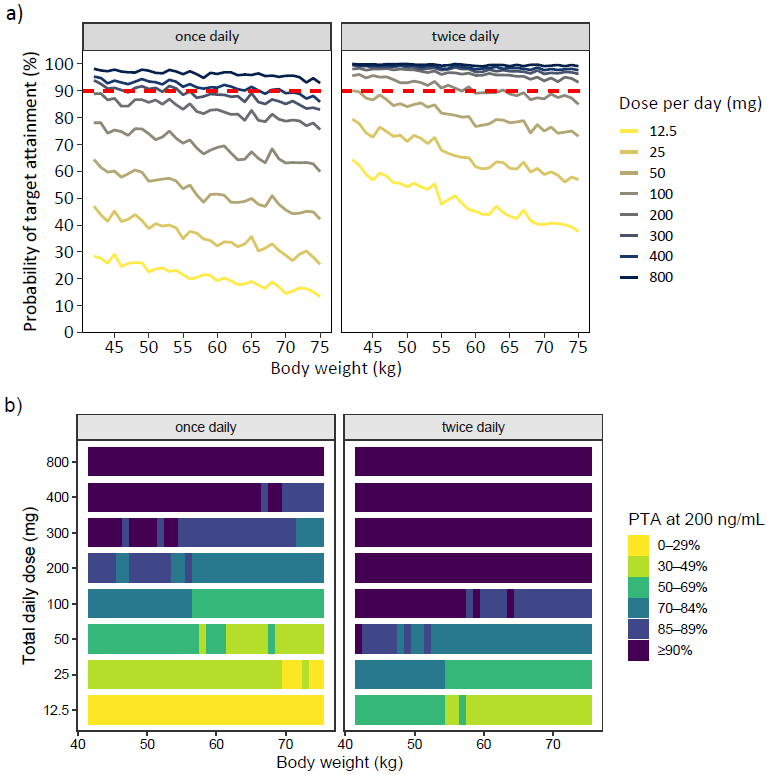


**Fig S6.** **Probability of target attainment across body weights at a target concentration of 200 ng/mL.**

a) PTA as a function of body weight for once-daily (left) and twice-daily (right) dosing regimens across daily oxfendazole doses of 12.5 to 800 mg. Each line represents a different total daily dose, either administered once daily (left), or split into two administrations (right panel). The horizontal red line indicates the target PTA of 90%. Simulations were performed for 1,000 adults per dosing regimen.

(b) Corresponding heat map representation of PTA at 200 ng/mL across body weights. Color coding indicates PTA categories as shown in the legend.


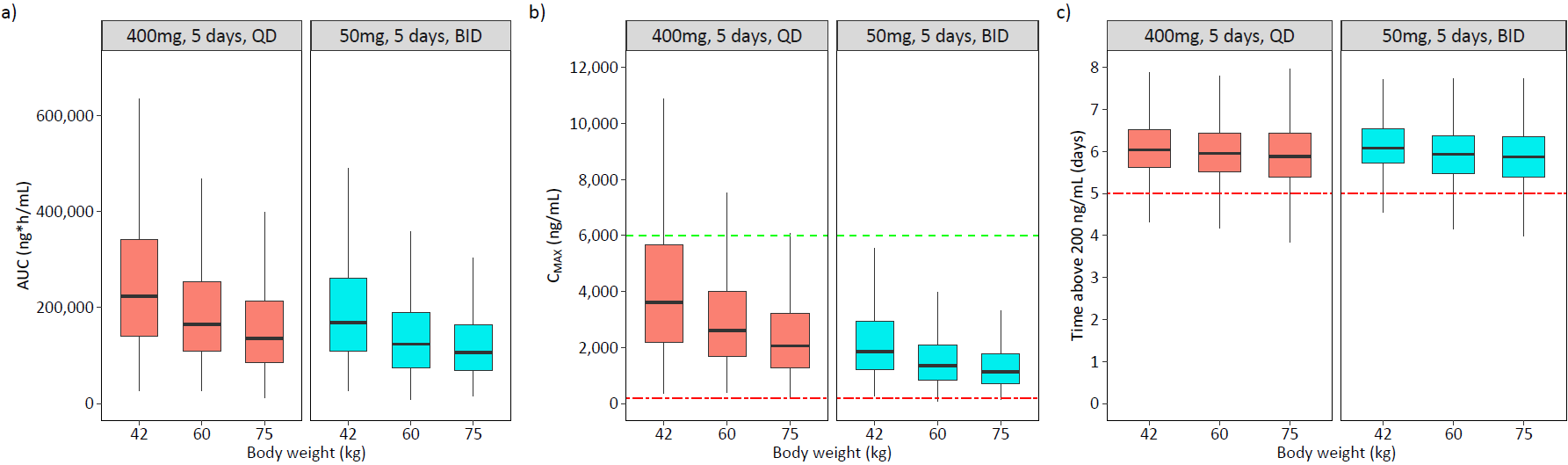
**Fig S7.** **Simulated oxfendazole plasma exposure stratified by body weight for selected dosing regimens.**

Comparison of once-daily dosing (400 mg QD) with twice-daily dosing (50 mg BID) administered for 5 days. Boxes represent the interquartile range (IQR), bold lines the median, whiskers 1.5×IQR. **a)** AUC_∞_, **b)** C_MAX_. **c)** Time above the target concentration (C_TARGET_ = 200 ng/mL. In panel b, the horizontal red line marks the putative target concentration; the horizontal green line shows a well-tolerated median C_MAX_ from a previous MAD study (15 mg/kg oxfendazole). In panel c, the horizontal red line represents the minimum target duration above C_TARGET_ (5 days).


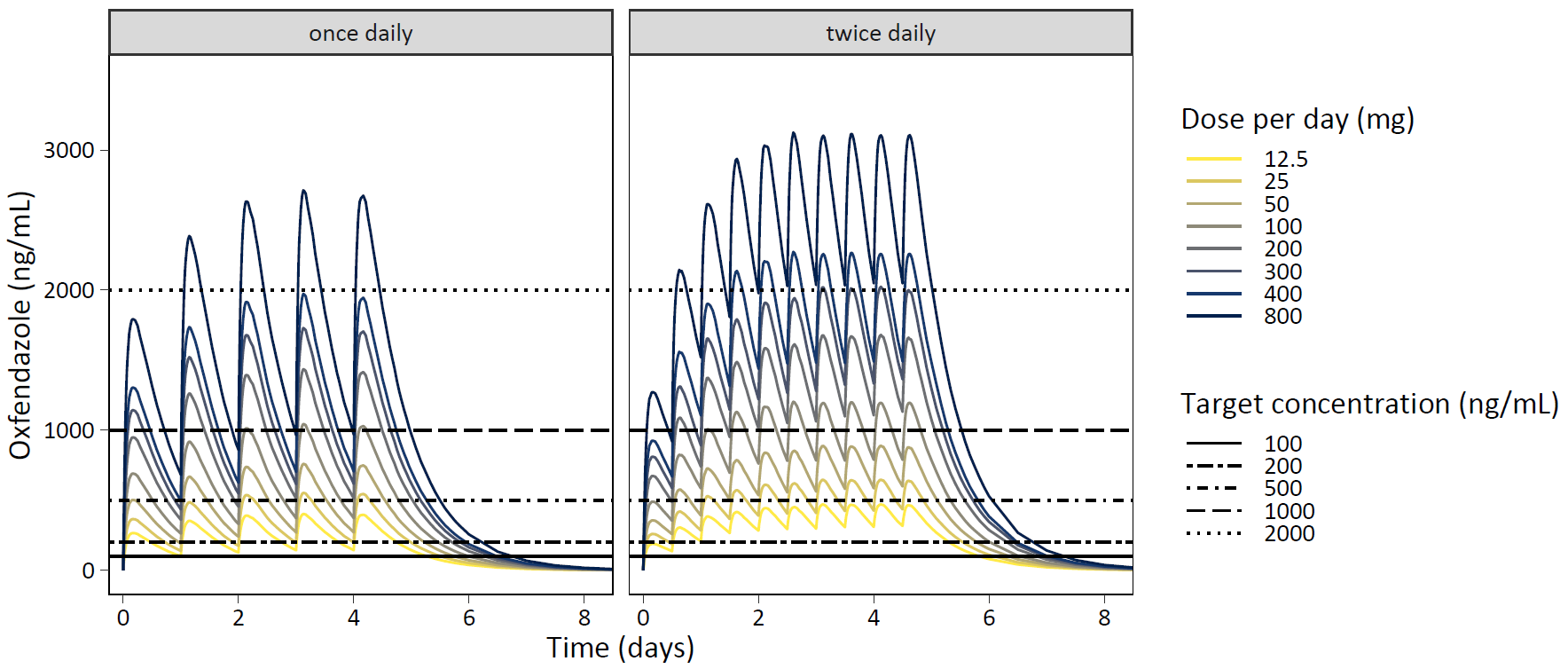


**Fig S8.** **Simulated oxfendazole PK profiles for once-daily and twice-daily dosing regimens across target concentrations.**

Simulated median concentration-time profiles of oxfendazole in human plasma following 5 days of dosing with 12.5 to 800 mg daily doses of oxfendazole, given either as a single daily dose (left panel) or split into two daily doses (right panel). PK profiles are shown for a range of target concentrations (100 to 2000 ng/mL).


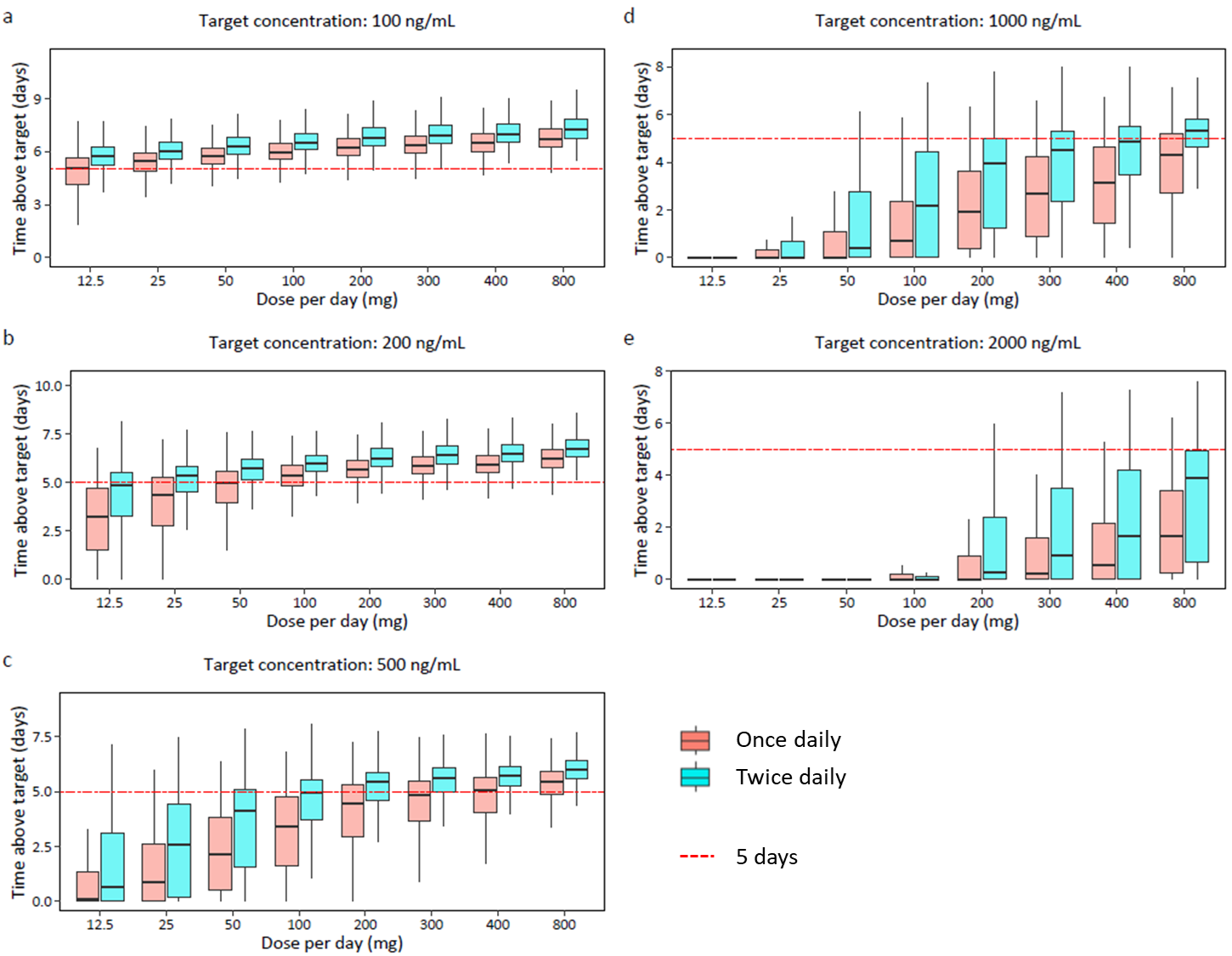


**Fig S9.** **Simulated oxfendazole time above target concentration for once-daily and twice-daily dosing regimens across target concentrations.**

Simulated oxfendazole plasma exposure (times above the target concentration) for different dosing regimens and a range of target concentrations (100 to 2000 ng/mL). Simulations were conducted for a 60 kg adult and two dosing strategies: once daily dosing, and splitting the daily dose into two administrations. Daily doses ranged from 12.5 to 800 mg oxfendazole for 5 days.

Boxplots show the distribution of simulated exposures; midline = median, box = interquartile range, whiskers = 1.5×IQR. The horizontal red line represents the minimum duration above the target concentration (5 days).

**Code S1**

NONMEM code of the final population PK model

;; Modeler: Frauke Assmus, Ayorinde Adehin, Richard Hoglund, Joel Tarning

;; Project name: Oxfendazole, Pooled Analysis

;;---------------------------------------------------------------------------------------------------------------------------------------------------------------------------------------------;;

$INPUT

ID ; Subject identifier

COHORT ; Cohort (1,2,3)

DAT1=DROP ; Analysis date

TIME ; Analysis time

DRG ; 1 oxfendazole (parent), 2 fenbendazole (M1 metabolite), 3 oxfendazole sulfone (M2 metabolite)

EVID ; 1 = dose, 0 = observation

BQL ; 0 = above LLOQ or dose, 1= below LLOQ

MDV ; 0 = not missing, 1= dose or missing

DV ; ln of dependent variable (nmol/L)

AMT ; Dose amount (nmol)

CMT ; Compartment (1 = dose, 2 = central oxfendazole, 3 = central fenbendazole, 4 = central oxfendazole sulfone)

WT ; Body weight (kg)

DOSE ; Dose (mg)

OCC ; OCC = 1 ; Cohort 3: OCC 1,2,3,4,5 (dosing occasion)

;;---------------------------------------------------------------------------------------------------------------------------------------------------------------------------------------------;;

$DATA

Input.csv ; Data input file

;;---------------------------------------------------------------------------------------------------------------------------------------------------------------------------------------------;;

$SUBROUTINE ADVAN5 TRANS1

;;---------------------------------------------------------------------------------------------------------------------------------------------------------------------------------------------;;

$MODEL

COMP=(1) ; Absorption compartment

COMP=(2) ; Central compartment (oxfendazole = parent)

COMP=(3) ; Central compartment (metabolite 1 = fenbendazole)

COMP=(4) ; Central compartment (metabolite 2 = oxfendazole sulfone)

;;---------------------------------------------------------------------------------------------------------------------------------------------------------------------------------------------;;

$PK

; Inter-occasion variability

OCC1 = 0

OCC2 = 0

OCC3 = 0

OCC4 = 0

OCC5 = 0

IF(OCC.EQ.1) OCC1=1 ; Dosing Occasion 1

IF(OCC.EQ.2) OCC2=1 ; Dosing Occasion 2

IF(OCC.EQ.3) OCC3=1 ; Dosing Occasion 3

IF(OCC.EQ.4) OCC4=1 ; Dosing Occasion 4

IF(OCC.EQ.5) OCC5=1 ; Dosing Occasion 5

IOV1 = 0

IOV1 = ETA(11)*OCC1 +ETA(12)*OCC2 +ETA(13)*OCC3 +ETA(14)*OCC4 +ETA(15)*OCC5

IOV2 = 0

IOV2 = ETA(16)*OCC1 +ETA(17)*OCC2 +ETA(18)*OCC3 +ETA(19)*OCC4 +ETA(20)*OCC5

; Dose effect

COV = (DOSE/34.7)**(-0.541)

;;---------------------------------------------------------------------------------------------------------------------------------------------------------------------------------------------;;

; Disposition parameters

TVCL_P = THETA(1) *((WT/70)**0.75) ; Elimination clearance (parent)

CL_P = TVCL_P*EXP(ETA(1))

TVV_P = THETA(2) *((WT/70)**1.00) ; Central volume (parent)

V_P = TVV_P*EXP(ETA(2))

TVKA = THETA(3) ; Absorption rate

KA = TVKA*EXP(ETA(3)+IOV2)

TVF1 = THETA(4) *COV ; Relative bioavailability

F1 = TVF1*EXP(ETA(4)+IOV1)

TVCL_M1=THETA(5) *((WT/70)**0.75) ; Elimination clearance (metabolite 1, fenbendazole)

CL_M1=TVCL_M1*EXP(ETA(5))

TVV_M1=THETA(6) *((WT/70)**1.00) ; Central volume (metabolite 1)

V_M1=TVV_M1*EXP(ETA(6))

TVFM = THETA(7) ; Fraction metabolized P --> M1

FM=EXP(LOG(TVFM/(1-TVFM))+ETA(7))/(1+EXP(LOG(TVFM/(1-TVFM))+ETA(7)))

TVCL_M2=THETA(8) *((WT/70)**0.75) ; Elimination clearance (metabolite 2, oxfendazole sulfone)

CL_M2=TVCL_M2*EXP(ETA(8))

TVV_M2=THETA(9) *((WT/70)**1.00) ; Central volume (metabolite 2)

V_M2=TVV_M2*EXP(ETA(9))

TVFFP = THETA(10) ; Fraction of the absorbed oxfendazole converted into oxfendazole sulfone during first-pass metabolism

FFP = EXP(LOG(TVFFP/(1-TVFFP))+ETA(10))/(1+EXP(LOG(TVFFP/(1-TVFFP))+ETA(10)))

K12 = KA*(1-FFP)

K14 = KA*(FFP)

K23 = FM*CL_P/V_P

K24 = (1-FM)*CL_P/V_P

K30 = CL_M1/V_M1

K40 = CL_M2/V_M2

S2 = V_P

S3 = V_M1

S4 = V_M2

;;---------------------------------------------------------------------------------------------------------------------------------------------------------------------------------------------;;

$ERROR

IF(CMT.EQ.2) IPRED = A(2)/S2 ; oxfendazole individual predictions

IF(CMT.EQ.2.AND.IPRED.GT.0) IPRED = LOG(IPRED) ; log transformed individual predictions

IF(CMT.EQ.2) W = SQRT(SIGMA(1,1)) ; weighting term

IF(CMT.EQ.2) Y = IPRED + EPS(1) ; model predicted observed plasma concentration

IF(CMT.EQ.3) IPRED = A(3)/S3 ; fenbendazole

IF(CMT.EQ.3.AND.IPRED.GT.0) IPRED = LOG(IPRED)

IF(CMT.EQ.3) W = SQRT(SIGMA(2,2))

IF(CMT.EQ.3) Y = IPRED + EPS(2)

IF(CMT.EQ.4) IPRED = A(4)/S4 ; oxfendazole sulfone

IF(CMT.EQ.4.AND.IPRED.GT.0) IPRED = LOG(IPRED)

IF(CMT.EQ.4) W = SQRT(SIGMA(3,3))

IF(CMT.EQ.4) Y = IPRED + EPS(3)

IRES = DV-IPRED ; Individual residual

IWRES = IRES/W ; Individual weighted residual

IF(AMT.GT.0) DTIM=TIME

TAD=TIME-DTIM ; calculating time after dose

;;---------------------------------------------------------------------------------------------------------------------------------------------------------------------------------------------;;

$THETA

(0, 3.54) ; 1 CL_OXF_/F (L/h)

(0, 67.2) ; 2 V_OXF_/F (L)

(0, 0.695) ; 3 K_A_ (h^-1^)

(1) FIX ; 4 F1

(0, 94.4) ; 5 CL_FEN_/F (L/h)

(0, 3370) ; 6 V_FEN_/F (L)

(0.5) FIX ; 7 f_M_

(0, 15.8) ; 8 CL_OXF-SO2_/F (L/h)

(0, 206) ; 9 V_OXF-SO2_/F (L)

(0, 0.0549,1) ; 10 f_FP_

;;---------------------------------------------------------------------------------------------------------------------------------------------------------------------------------------------;;

$OMEGA ; as variances

$OMEGA 0.0161 ; IIV_CL

$OMEGA 0.0387 ; IIV_V2

$OMEGA 0.924 ; IIV_KA

$OMEGA 0.424 ; IIV_F1

$OMEGA 0.618 ; IIV_CL_M1

$OMEGA 0.569 ; IIV_V_M1

$OMEGA 0 FIX ; IIV_FM

$OMEGA 0.0552 ; IIV_CL_M2

$OMEGA 0 FIX ; IIV_V_M2

$OMEGA 0.192 ; IIV_FFP

$OMEGA BLOCK(1) 0.141 ; IOV F1

$OMEGA BLOCK(1) SAME

$OMEGA BLOCK(1) SAME

$OMEGA BLOCK(1) SAME

$OMEGA BLOCK(1) SAME

$OMEGA BLOCK(1) 0.257 ; IOV KA

$OMEGA BLOCK(1) SAME

$OMEGA BLOCK(1) SAME

$OMEGA BLOCK(1) SAME

$OMEGA BLOCK(1) SAME

;;---------------------------------------------------------------------------------------------------------------------------------------------------------------------------------------------;;

$SIGMA ; as variances

$SIGMA 0.0231 ; Residual error parent (oxfendazole)

$SIGMA 0.177 ; Residual error metabolite 1 (fenbendazole)

$SIGMA 0.0189 ; Residual error metabolite 2 (oxfendazole sulfone)

;;---------------------------------------------------------------------------------------------------------------------------------------------------------------------------------------------;;

$ESTIMATION

MAXEVAL=9999 PRINT=5 METHOD=1 INTER MCETA=50

**Code S2**

R code for the computation of CV% for fraction of first-pass metabolism, f _FP_

theta_med <- 0.0549 # median of f _FP_ , representing the typical value of the population in the normal scale

omega_bio <- 0.192 # variance of ETA for f _FP_, representing inter-individual variability on the logit scale

sd_bio <- sqrt(omega_bio) # Standard deviation on the logit scale

theta_bio <- log(theta_med / (1 - theta_med)) # Computation of the mean f _FP_ on the logit scale

logit_sample <- rnorm(10^7, theta_bio, sd = sd_bio) # sampling step from the logit distribution

indiv_bios <- 1 / (1 + 1/exp(logit_sample)) # individual values for f_FP_, representing IIV in the normal scale

expected_cv <- 100 * (sd(indiv_bios) / mean(indiv_bios)) # expected CV% using normal-scale values for f _FP_

expected_cv

Notably, f_FP_ (fraction of first-pass metabolism) was modeled on the logit scale to maintain biologically plausible values between 0 and 1. For logit-transformed parameters, no analytical solution exists to directly convert variances into CV%. Hence, we computed the CV% for f_FP_ via numerical simulation, where random ETA values were sampled from the logit scale, back-transformed to the natural scale, and used to compute CV% as 100 × (SD/mean). This approach provides an approximate CV% and is easily interpretable, but may not fully capture the skewness of the true distribution. For transparency and reproducibility, the underlying variance values are provided in **Supplementary Code S1**.

**Reference:**

Prybylski JP. Reporting Coefficient of Variation for Logit, Box-Cox and Other Non-log-normal Parameters. Clinical pharmacokinetics. 2024;63(2):133-5.
